# New criteria for selecting the origin of DNA replication in *Wolbachia *and closely related bacteria

**DOI:** 10.1186/1471-2164-8-182

**Published:** 2007-06-20

**Authors:** Panagiotis Ioannidis, Julie C Dunning Hotopp, Panagiotis Sapountzis, Stefanos Siozios, Georgios Tsiamis, Seth R Bordenstein, Laura Baldo, John H Werren, Kostas Bourtzis

**Affiliations:** 1 Department of Environmental and Natural Resources Management, University of Ioannina, 2 Seferi Street, Agrinio 30100, Greece; 2 The Institute for Genomic Research, J. Craig Venter Institute, 9712 Medical Center Drive, Rockville, MD 20850, USA; 3 Josephine Bay Paul Center for Comparative Molecular Biology and Evolution, The Marine Biological Laboratory, Woods Hole, MA, 02543, USA; 4 Department of Biology, University of California, Riverside, USA; 5 Department of Biology, University of Rochester, Rochester, USA

## Abstract

**Background:**

The annotated genomes of two closely related strains of the intracellular bacterium *Wolbachia pipientis *have been reported without the identifications of the putative origin of replication (*ori*). Identifying the *ori *of these bacteria and related alpha-Proteobacteria as well as their patterns of sequence evolution will aid studies of cell replication and cell density, as well as the potential genetic manipulation of these widespread intracellular bacteria.

**Results:**

Using features that have been previously experimentally verified in the alpha-Proteobacterium *Caulobacter crescentus*, the origin of DNA replication (*ori*) regions were identified *in silico *for *Wolbachia *strains and eleven other related bacteria belonging to *Ehrlichia*, *Anaplasma*, and *Rickettsia *genera. These features include DnaA-, CtrA- and IHF-binding sites as well as the flanking genes in *C. crescentus*. The *Wolbachia ori *boundary genes were found to be *hemE *and COG1253 protein (CBS domain protein). Comparisons of the putative *ori *region among related *Wolbachia *strains showed higher conservation of bases within binding sites.

**Conclusion:**

The sequences of the *ori *regions described here are only similar among closely related bacteria while fundamental characteristics like presence of DnaA and IHF binding sites as well as the boundary genes are more widely conserved. The relative paucity of CtrA binding sites in the *ori *regions, as well as the absence of key enzymes associated with DNA replication in the respective genomes, suggest that several of these obligate intracellular bacteria may have altered replication mechanisms. Based on these analyses, criteria are set forth for identifying the *ori *region in genome sequencing projects.

## Background

*Wolbachia *are Gram-negative, intracellular α-Proteobacteria that infect many invertebrates including terrestrial crustaceans, mites, spiders and filarial nematodes [1-4]. Much of the success of *Wolbachia *can be attributed to the diverse phenotypes they induce in hosts. These range from classical mutualism to reproductive parasitism as characterized by the ability to override chromosomal sex determination, induce parthenogenesis, selectively kill males and induce cytoplasmic incompatibility in early embryos [2-4]. The unique biology of *Wolbachia *has attracted a growing number of researchers interested in questions ranging from the evolutionary implications of infection to the use of this agent for pest and disease control [5-9].

These endosymbiotic bacteria are typically transmitted through the eggs of their hosts and their replication rate is regulated to avoid overgrowth prior to host reproduction [2-4]. The replication control mechanisms are not known for this growth limitation. The intensity of *Wolbachia*'s effects has often been correlated with bacterial copy number, as reported in different host species [10-13]. A factor that may influence bacterial proliferation is the organization of the *ori *region. Identifying the *ori *of these bacteria is a key step in understanding the mechanisms of bacterial replication and for developing methods for genetic manipulation of these bacteria. Recently, the closed and annotated genomes of two *Wolbachia *strains have been reported [14,15]. However, neither of these studies identified the putative origin of replication (*ori*) for *Wolbachia*.

DNA replication in bacteria takes place by uncoiling the double stranded helix and breaking the hydrogen bonds between the complementary strands at a specific chromosomal locus, the *ori *region. Early events in DNA replication are subdivided into the following three steps: (i) binding of the initiator proteins to sites located within the *ori *region; (ii) local unwinding of the *ori *region; and (iii) loading of the DNA helicase and other proteins required to form the Y-shaped replication forks [16]. Typically, bacteria have a single *ori *region [17], although in some prokaryotes two chromosomal *ori *regions were experimentally identified, probably reflecting a temporal mode of DNA replication [18,19].

Chromosomal replication initiates at *ori*, proceeds bidirectionally and terminates when the replication forks reach the termination site, *terC *(in the case of circular chromosomes) or the chromosome ends (in the case of linear chromosomes) [20]. The initiation of bacterial chromosome replication is mediated by the DnaA protein, which binds to specific 9-mer cis-regulatory elements called DnaA boxes located in the *ori *region. Usually, about ten to twenty DnaA molecules bind to five DnaA boxes and promote unwinding of the AT-rich *ori *region. The sequences of *ori *are conserved only among closely related microorganisms and vary greatly in size (from 200–1000 bp) [17]. A common feature is the presence of several DnaA boxes and an AT-rich region; a cluster of four or more DnaA boxes is indicative of a functional origin of replication [17]. In γ-Proteobacteria, the *ori *region is frequently located within the *rnpA-rmpH-dnaA-dnaN-recF-gyrB *gene cluster and usually next to the *dnaA *gene [17], with the *Escherichia coli ori *being the most thoroughly studied [21,22].

The location of the *ori *region can be diverse across the bacterial lineages [23]. Among the α-Proteobacteria, which includes *Wolbachia*, only the *ori *region of *C. crescentus *(*Cori*) has been experimentally identified [24]. Independent methods have provided a consistent location for *Cori *between *hemE *(CC3763) and a gene encoding a conserved hypothetical protein (CC0001) (in the present study, this gene is referred to by its NCBI COG number, COG1806) [24]. The *hemE *gene encodes for uroporphyrinogen decarboxylase – a component in heme biosynthesis, and the COG1806 gene has no known function but contains the conserved domain of unknown function, DUF299. The *hemE*/COG1806 boundary genes of *Cori *are present in the sequenced genomes of several other α-Proteobacteria [25]. While *Cori *shows some apparent similarities with the *E. coli ori *such as a 40 bp AT-rich region, presence of DnaA boxes and an IHF (integration host factor) binding site, it has an additional regulatory protein, CtrA. CtrA is a global cell cycle regulator that controls 26% of the transcripts that vary during the cell cycle [26]. *In vitro *footprint experiments revealed five CtrA binding sites within *Cori*, centered over the consensus TTAA-N_7_-TTAA [27]. CtrA binding sites appear to be strategically organized spanning the entire length of *Cori *[24]. Phosphorylated CtrA binds two perfect or imperfect halves of the recognition sequence, probably as a dimer and represses chromosomal replication.

Comparison of over 30 bacterial genomes with *Cori *reveals a noticeable conservation of binding sites and flanking genes, although in some bacteria, chromosomal rearrangements appear to have taken place (unpublished observations). GC-skew analysis has also been used to predict the origin of DNA replication [17,28-32], but it is not a good universal predictor of the *ori *[33], and was not predictive for the two annotated *Wolbachia *genomes [14,15]. In contrast, for the closely related *Ehrlichia*, *Neorickettsia*, and *Anaplasma*, GC-skew has been used to approximate the origin position in the chromosome more reliably [34-36]. However, the actual shift can occur over a fairly large region and thus this feature alone cannot reveal the precise location for the ori region.

Given that GC-skew analysis was insufficient at predicting the *ori *region in several α-Proteobacteria species, a different approach was developed. The origin of replication in *C. crescentus *is located between orthologs of *hemE *(CC_3763) and COG1806 (CC_0001), and it contains five DnaA boxes, a single binding site for IHF and five CtrA binding sites [37]. In *Rickettsia prowazekii*, the *ori *region is also located between the *hemE *(RP_885) and COG1806 orthologs [37].

The guidelines used by Brassinga et al. [37] for detecting *Cori *were applied in the present study to identify the origin of DNA replication in *Wolbachia *and in ten closely related α-Proteobacteria. Computational analyses indicate that the origin of DNA replication in the *w*Mel and *w*Bm *Wolbachia *strains [14,15] lies between a gene encoding a cystathione-β-synthase (CBS) domain protein and the *Wolbachia hemE *gene. The evidence relies mainly on boundary gene recognition as well as DnaA-, CtrA- and IHF-binding site identification, as described by [37]. Analysis of the corresponding sequences from an additional fifty-one *Wolbachia *strains supports the predicted *ori *as well as identifying frequent recombination at the edges of the sequences. Using the same guidelines, the *ori *region was also identified in the sequenced representatives of the closely related *Anaplasma*, *Ehrlichia*, *Neorickettsia *and *Rickettsia*.

## Results

### *In silico *prediction of the *Wolbachia *origin of replication

The origin of replication of *C. crescentus *is located between orthologs of *hemE *(CC_3763) and a conserved hypothetical protein, COG1806, and contains DnaA, CtrA and IHF binding sites [37]: In *R. prowazekii*, the *ori *region is also located between the *hemE *(RP_885) and COG1806 orthologs.

Based on a slightly modified approach and findings of Brassinga et al. [37], the *Wolbachia ori *was predicted *in silico*. Our approach was based on four criteria: (a) position near either *hemE *or COG1806 orthologs; (b) an intergenic region (that is, the putative *ori *region) containing an appropriate number of binding sites for the DnaA, CtrA and IHF factors; (c) genome-wide searches confirming that no other appropriately sized intergenic region (>300 bp) contains a significant number of these three characteristic binding sites and (d) the AT-content of the predicted origins that is higher than the average for the respective genome.

First, the *Wolbachia *orthologs of *hemE *and COG1806 were searched in the recently published *Wolbachia w*Mel genome [15] and identified as the loci WD_1028 and WD_0341, respectively. The great distance between the two genes in *Wolbachia *(>600 kb) indicated no conservation of the *hemE*-COG1806 region in *Wolbachia*. The lack of conservation is most likely due to a single chromosomal rearrangement resulting in two possible locations for *ori*: WD_0340-WD_0341 or WD_1027-WD_1028. The first region is between the heme exporter WD_0340 (*ccmC*) and the COG1806 homolog (WD_0341). The second region is between the CBS domain protein WD_1027 (referred to in the present study after its NCBI COG number, COG1253) and uroporphyrinogen decarboxylase WD_1028 (*hemE*).

Both regions were examined for the presence of DnaA-, CtrA-, and IHF-binding sites, essential components of *ori *[37]. Only the intergenic region (IGR) between the COG1253 and *hemE *gene (position 988364–988765, 402 bp) was found to have all three characteristic binding sites. In addition, out of all 110 IGRs of greater than 300 bp, this region has the highest number of CtrA, DnaA and IHF binding sites (eight total, p = 1/110 = 0.009), the highest density of total binding domains per IGR bp (0.020 per bp). The next closest is the IGR between WD_0248-WD_0249, which has a much lower total number of binding sites (six) and binding site density (0.013 per bp). However, it lacks putative DnaA boxes, a prerequisite for an *ori *region. The putative *ori *region is one of only two IGRs > 300 bp that have all three binding site types (p = 2/110 = 0.018). However, the IGR between WD_0100-WD_0102 has only 4 total binding domains (compared to 8) and, due to it's larger size (545 bp) a binding site density 1/3 that of the putative ori (0.007 *vs *0.020). These data, combined with the association of the WD_1027-WD_1028 IGR with the same genes shown to be flanking the *ori *region in *C. crescentus *and *R. prowazekii*, provides compelling evidence that this is most likely, the origin of replication. More specifically, there are 3 DnaA, 4 CtrA and 1 IHF binding sites in the 404 bp region between COG1253 and *hemE*. In contrast, the region between *ccmC *and COG1806 has only a single IHF binding site and is only 130 bp long, which is rather short compared to the *ori *of related bacteria (e.g. *R. prowazekii *and *C. crescentus*, have replication origins >400 bp [37]).

Another feature of origins is that they typically contain an AT-rich region to facilitate dissociation of the DNA during replication initiation [17]. As a result, the overall AT-content of *ori *is higher when compared to the respective genome. Consistent with this feature, the putative *w*Mel origin is 76% AT-rich compared to the 65% average AT-content for the genome. In addition, the *ori *region is significantly more AT-rich than the average found for other intergenic regions of >300 bp. Of 110 intergenic regions >300 bp, only 6 have AT-content greater or equal to the *ori *(p = 6/110 = 0.055). Indeed, the AT-contents of all origins detected in this study were higher than their respective genome averages (Table 1). It has been proposed in *E. coli *that this exceptionally AT-rich DNA is the place where the initiator factor of DNA replication, the DnaA protein, first unwinds the origin [38,39]. These AT-rich sequences are also conspicuous because they are tandem repeats. For example, the *E. coli *oriC region is composed of three imperfect 13-mer sequences with the consensus sequence GATCTNTTNTTTT [38]. Similar tandem repeats were detected in all bacterial species and strains studied except in *A. marginale*, *E. canis *and *N. sennetsu*. In most cases the repeats were 10–12 bp long and appeared in two copies (data not shown).

**Table 1:** Bacterial species/strains whose partial and/or complete genome sequence was downloaded from various sources

Organism	References	Closed-Curated annotation	Genbank	Coordinates (length)	Flank One	Flank Two	% genome AT content	% *ori *AT content
*Wolbachia pipientis w*Mel_A	[15]	Y	NC_002978	988364..988765 (402 bp)	WD_1027/*COG1253 *(CBS)	WD_1028/*hemE*	64.77	75.87
*Wolbachia pipientis w*Bm_D	[14]	Y	NC_006833	1079740..110 (455 bp)	Wbm_0809/*COG1253 *(CBS)	Wbm_0001/*hemE*	65.82	69.89
*Wolbachia pipientis w*Ana_A	[43]	N	NZ_AAGB00000000	N/A (402 bp)	WwAna0355/*COG1253 *(CBS)	WwAna0353/*hemE*	N/A	75.87
*Wolbachia pipientis w*Sim_A	[43]	N	NZ_AAGC00000000	N/A (396 bp)	WwSim0491/*COG1253 *(CBS)	WwSim0492/*hemE*	N/A	75.51
*Wolbachia pipientis w*Wil_A	[44]	N	N/A	N/A (402 bp)	*COG1253 *(CBS)	*hemE*	N/A	75.49
*Wolbachia pipientis w*Pip_B	[45]	N	N/A	N/A (402 bp)	*COG1253 *(CBS)	*hemE*	N/A	79.17
*Ehrlichia chaffeensis *Arkansas	[36]	Y	CP000236	26939..27361 (423 bp)	ECH_0030/*hemE*	ECH_0031/*COG1253*	69.90	74.70
*Ehrlichia ruminantium *Welgevonden	[35]	Y	NC_005295	23003..23424 (422 bp)	Erum_0180/*hemE*	Erum_0190/*COG1253*	72.52	75.19
*Ehrlichia ruminantium *Gardel	[88]	Y	NC_006831	4149..4564 (416 bp)	ERGA_CDS_00050/*hemE*	ERGA_CDS_00060/*COG1253*	72.49	75.48
*Ehrlichia canis *Jake	NC_007354	Y	NC_007354	11395..11819 (425 bp)	Ecaj_0010/*hemE*	Ecaj_0011/*COG1253*	71.04	73.88
*Anaplasma marginale *St. Maries	[34]	Y	NC_004842	1147944..1148563 (620 bp)	AM_1301/*hemE*	AM_1303/*COG1253*	50.24	63.71
*Anaplasma phagocytophilum *HZ	[36]	Y	CP000235	20557..20941 (385 bp)	APH_0020/*hemE*	APH_0021/*COG1253*	58.36	71.43
*Neorickettsia sennetsu *Miyayama	[36]	Y	CP000237	855136..855541 (406 bp)	NSE_0967/phage uncharacterized protein	NSE_0968/*hemE*	58.92	71.92
	[36]	Y	CP000237	267777..268329 (553 bp)	NSE_0324/hypothetical protein	NSE_0327/*COG1253*	58.92	58.22
*Rickettsia typhi *Wilmington	[75]	Y	NC_006142	1111114..1111496 (383 bp)	RT_0877/*hemE*	RT_0001/*COG1806*	71.08	82.25
*Rickettsia prowazekii *Madrid E	[73]	Y	NC_000963	1111140..1111523 (384 bp)	RP_885/*hemE*	RP_001/*COG1806*	71.00	82.55
*Rickettsia conorii *Malish 7	[74]	Y	NC_003103	1268361..1268755 (395 bp)	RC_1374/*hemE*	RC_0001/*COG1806*	67.56	81.52
*Rickettsia felis *URRWXCal2	[76]	Y	NC_007109	1484753..1485148 (396 bp)	RF_1400/*hemE*	RF_0001/*COG1806*	67.55	82.83
*Caulobacter crescentus *CB15	[89]	Y	NC_002696	4016703..159 (404 bp)	CC_3763/*hemE*	CC_0001/*COG1806*	32.80	43.56

Y, yes; N, no; N/A, not applicable.

These lines of *in silico *evidence suggest that *Wolbachia *has an origin of replication on its chromosome that lies between the COG1253 and *hemE *genes. One of the boundary genes, *hemE *(WD_1028), encodes for uroporphyrinogen decarboxylase – a component in heme biosynthesis. The other, COG1253 (WD_1027), encodes for a CBS domain protein. CBS domains have been shown to bind ligands with an adenosyl group such as AMP, ATP and S-AdoMet [40]. Using the publicly available HMMer program [41], it was found that this protein has a CBS domain pair and a CorC_HlyC transporter associated domain.

**Figure 1:**
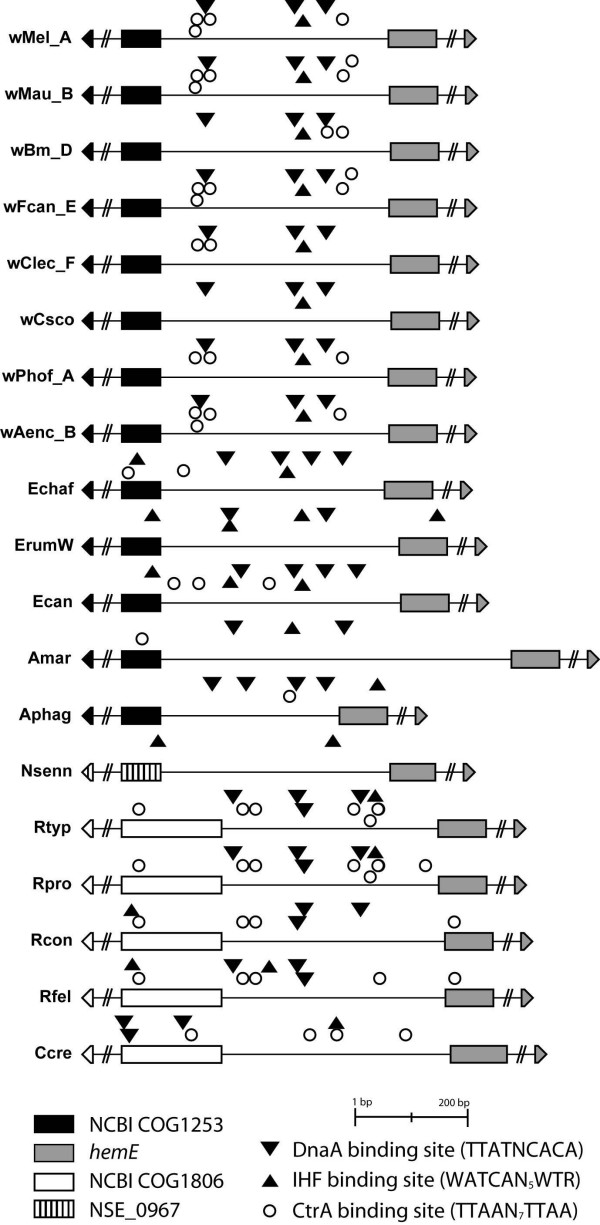
**Schematic representation of representative origins of replication**. Schematic drawing of *ori *regions from *Wolbachia*, *Anaplasma*, *Ehrlichia *and *Rickettsia*. Inverted triangles denote DnaA boxes, circles denote CtrA binding sites and triangles indicate IHF binding sites. The flanking genes are fragmented. Some protein binding sites are located outside *ori *but within either of the boundary genes; however, the flanking genes were not fully sequenced from the *Wolbachia *strains of *F. candida*, *C. lectularius*, *C. scorpioides*, *P. hoffmeyeri*, and *A. encedon*. Note also that in *A. marginale *the *ori *region appears to be significantly longer due to the differently annotated *hemE *gene (see text for details). *w*Mel_A, *Wolbachia *of *Drosophila melanogaster w*Mel; *w*Mau_B, *Wolbachia *of *Drosophila mauritiana w*Mau; *w*Bm_D, *Wolbachia *of *Brugia malayi w*Bm; *w*Fcan_E, *Wolbachia *of *Folsomia candida w*FcanE; *w*Clec_F, *Wolbachia *of *Cimex lectularius w*ClecF; *w*Csco, *Wolbachia *of *Cordylochernes scorpioides w*Csco; *w*Phof_A, *Wolbachia *of *Pegoscapus hoffmeyeri w*PhofA; *w*Aenc_B, *Wolbachia *of *Acraea encedon w*AencB; Echaf, *Ehrlichia chaffeensis *Arkansas; ErumW, *E. ruminantium *Welgevonden; Ecan, *E. canis *Jake; Amar, *Anaplasma marginale *St Maries; Aphag, *A. phagocytophilum *HZ; Nsenn, *Neorickettsia sennetsu *Miyayama; Rtyp, *Rickettsia typhi *Wilmington; Rpro, *R. prowazekii *Madrid E; Rcon, *R. conorii *Malish 7; Rfel, *R. felis *URRWXCal2; Ccre, *Caulobacter crescentus *CB15.

The putative *ori *regions were also identified in other complete and/or partial publicly available *Wolbachia *genomes by using the above mentioned criteria. The orthologs of the COG1253 (WD_1027) and *hemE *(WD_1028) genes were identified by BLAST searches [42] of the genomes of the *w*Ana [43], *w*Sim [43], *w*Wil [44], *w*Pip [45] and *w*Bm *Wolbachia *strains [14] (see Table 1). Both flanking genes are highly conserved in position and in sequence. Based on criteria set forth by Zyskind et al. [46], the regions of the *w*Mel and *w*Bm strains for which their genome sequence is available [14,15], were compared indicating that: (a) 266 of 347, or 77%, of the amino acids are conserved, while 790 of 1044 (76%) of the nucleotides are conserved in the flanking gene *hemE *and (b) 216 of 279, or 77%, of the amino acids are conserved, while 609 of 819 (74%) of the nucleotides are conserved in the flanking gene COG1253. The *ori *regions of the two genomes present 73% identity at the nucleotide level. The putative *ori-*region-related binding sites for the *w*Ana, *w*Sim and *w*Wil strains (all members of the A-supergroup), *w*Pip strain (B-supergroup), and the *w*Bm strain (D-supergroup) have been depicted [see Additional Table 3]. A schematic representation is depicted in Figure 1 while the length and genome coordinates are shown in Table 1 (where applicable).

Based on the consensus sequence of the flanking genes of the putative *ori *region in *Wolbachia *[see Additional Text File 1], a PCR strategy [see Additional Figure 1 and Additional Table 2] was developed to amplify and sequence the putative *ori *regions of other *Wolbachia *strains infecting different *Drosophila *species: *w*MelPop, *w*Ri, *w*Au, *w*Ha, *w*Yak, *w*Tei, *w*San, *w*No, *w*Ma and *w*Mau [see Additional Table 1] [47-57]. Further sequencing of the putative *ori *region of an additional 37 *Wolbachia *strains from diverse hosts confirmed the overall characteristics of this region both in size and sequence [see Figure 1 and Additional Table 1]. *Wolbachia *strains belong to eight supergroups A-H [58-61]. The present study includes representative strains from supergroups A, B, D, E and F. All strains present three DnaA and a single IHF boxes while they differ in the number of CtrA binding sites (two to seven). The sequenced putative *ori *regions of some *Wolbachia *strains present a peculiar pattern of binding sites [see Figure 1 and Additional Table 1]. It is worth noting that the pseudoscorpion *Wolbachia ori *does not have any CtrA binding motifs while it presents three DnaA boxes and a single IHF binding site suggesting either the existence of a very diverged CtrA binding motif (which could not be identified when compared to the consensus sequence used in the present study) or the CtrA factor may not play an important regulatory role in the DNA replication of this *Wolbachia *strain.

**Figure 2:**
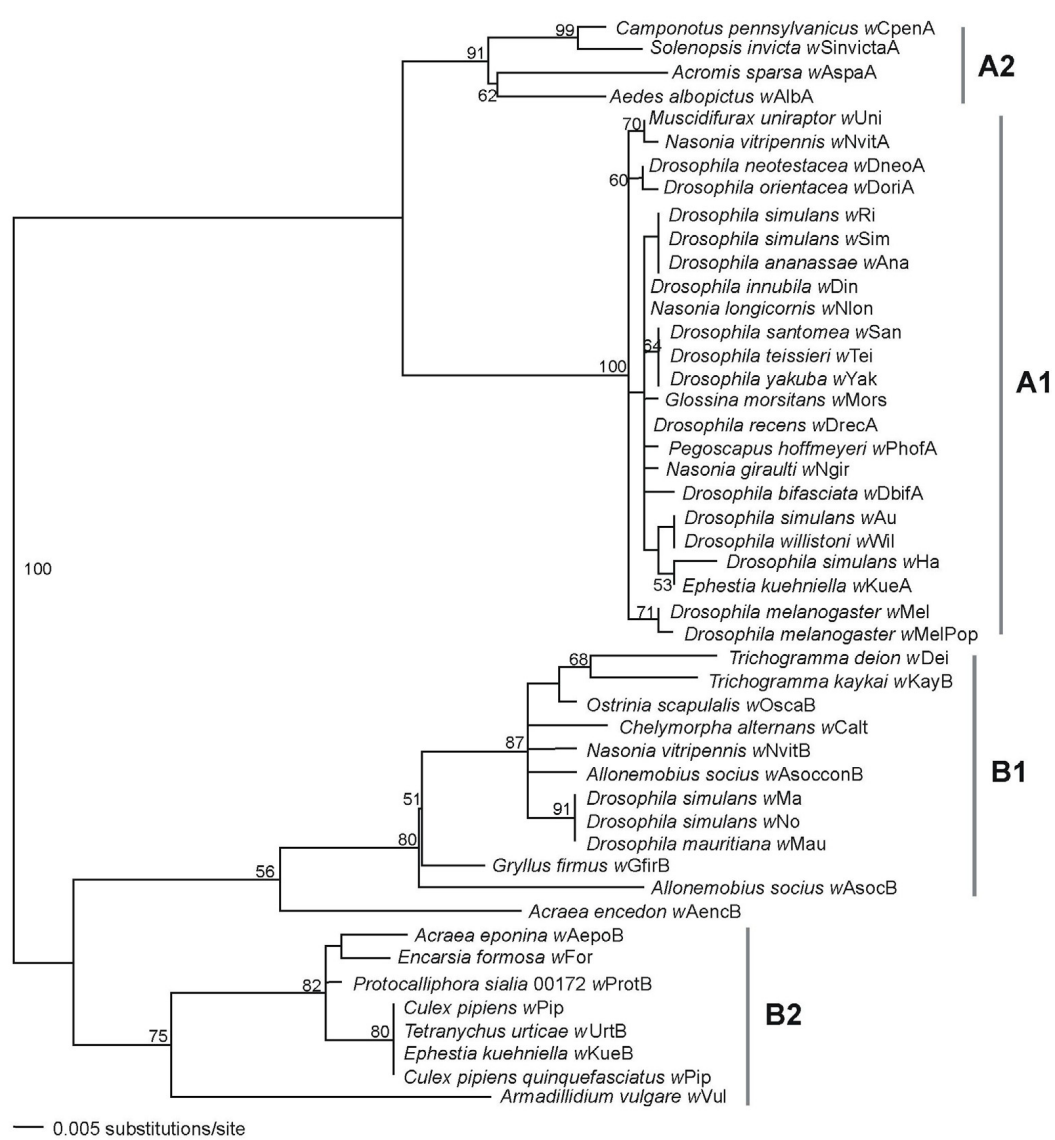
***ori *phylogeny based on 47 *Wolbachia *taxa**. Maximum Likelihood (ML) inferred phylogeny and ML bootstrap values based on the nucleotide sequences of the *ori *region from the A and B Wolbachia. Subgroups are denoted A1 and A2, and B1 and B2.

We tested for selective constraints on the binding sites for DnaA, CtrA and IHF by comparing the frequencies of polymorphic base positions in binding domain sites versus non-binding domain sites within the same IGR in different clades containing closely related *Wolbachia *strains (B1, B2 and A1, figure 2). The proportion of positions showing polymorphism was lower in the binding domain sites than non-binding sites for all three clades (B1 0.08 *vs *0.19, p = 0.035; B2 0 *vs *0.12, p = 0.001; A1 0.02 *vs *0.08, p = 0.081; Fisher Exact Test), with two showing significant reductions in polymorphisms within domain positions. Combining the data give 25.3 percent lower proportion of positions with polymorphisms in the binding domains relative to flanking non-binding positions (p < 0.0001 Fisher Exact Test), indicating some selective constraint on these positions.

### *In silico *prediction of the origin of DNA replication in closely related bacteria

Applying the same criteria, the origin of DNA replication was predicted *in silico *for the closely related *A. marginale *St. Maries, *A. phagocytophilum *HZ, *E. ruminantium *Welgevonden, *E. ruminantium *Gardel, *E. canis *Jake and *E. chaffeensis *Arkansas (Table 1). Annotation, BLAST analysis and/or domain searches (using HMMer) were used to identify the two flanking genes of the putative *ori *regions (Table 1).

The length of the predicted *ori *region differs markedly by 300 bp between the two *Anaplasma *spp [34,36]. In *A. marginale*, the *ori *is 620 bp; in *A. phagocytophilum *it is just 385 bp (Table 1). Both species have a single CtrA binding site and a single IHF binding site that differ in both sequence and position. *A. marginale *has two DnaA boxes while *A. phagocytophilum *has four [see Figure 1 and Additional Table 1].

Origin of replication features of the different *Ehrlichia *spp. examined in this study are summarized in Table 1. Multiple possible IHF binding sites are found for each member (depicted in Figure 1). Of special interest is the absence of CtrA binding sites in *E. ruminantium*. However, if two mismatches are allowed two putative CtrA sites are found.

The putative *ori *region of the four different *Rickettsia *spp. examined here was predicted by searching for the previously described *hemE*/COG1806 [37] [see Figure 1, Table 1 and Additional Table 1]. Following the same type of analysis, two putative *ori *regions can be identified in *N. sennetsu *that are unlike other bacteria examined in this study [see Table 1 and Additional Table 1]. The first region is located between COG1806 and COG1253, is 553 bp long, and has no DnaA, no CtrA and no IHF binding site. This intergenic region contains a hypothetical gene (92 amino acids long) and a predicted tRNA-Arg. The second region is located between *hemE *and an uncharacterized phage protein, is 408 bp long, and has no DnaA, no CtrA and two IHF binding sites. Although less clearly defined by the binding sites, this latter region is close to the shift in GC-skew [36] and its AT-content is higher when compared to the respective genome. Additionally, when it is compared with the first one, it appears to have more IHF binding sites in its immediate neighborhood. The above data indicate that the second region may be the putative *ori *region of *N. sennetsu *(Figure 1); however, it is possible that the proposed search criteria may not be appropriate for the identification of the origin of replication of *N. sennetsu*.

Taken together, the *in silico *approach can predict the putative origin of replication of most bacterial species closely related to *Wolbachia *including *Ehrlichia*, *Anaplasma*, and *Rickettsia*. In addition, the Maximum Likelihood derived trees demonstrate a clear concordance of the *ori *region and 16S rRNA phylogenies for the *Rickettsia *genus, *Anaplasma/Ehrlichia *genera, and *Wolbachia *supergroups [see Additional Figure 2].

### Recombination of the *Wolbachia ori *region

**Figure 3:**
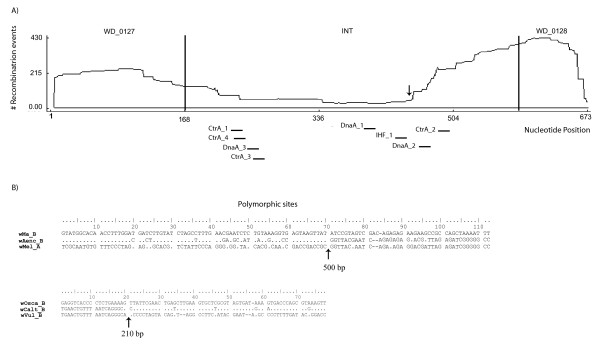
**Recombination within *Wolbachia ori *sequences**. Recombination results of the *Wolbachia ori *based on MaxChi (P < 0.001). (A) pattern of distribution of recombination breakpoints along the nucleotide alignment of 38 strains belonging to supergroup A and B. The cumulative number of recombination events per site is given. The alignment includes the beginning of locus *COG1253 *(WD1027, 1–168 bp), the intergenic non-coding region (INT, 169–586 bp), and the beginning of locus *hemE *(WD1028, 587–673 bp). Most of the breakpoints fall at the two edges of the intergenic region. Location of binding sites in the intergenic region is also shown. CtrA_1: 225-239 bp; CtrA_2: 489-503 bp (complementary strand); CtrA_3: 251-265 bp; CtrA_4: 228-242 (complementary strand); DnaA_1: 406-414 bp; DnaA_2: 461-469 bp; DnaA_3: 246-254 bp (complementary strand); IHF_1: 430-442 bp. Arrow indicates a major breakpoint. (B) Two examples of recombination among strains within supergroup B and between supergroup A and B. Only polymorphic sites are shown. For each example, putative recombination breakpoints are predicted based on the difference of shared polymophisms among the three strains and are indicated with an arrow (corresponding position in the nucleotide alignment is given). The *w*Aenc_B *Wolbachia ori *sequence (belonging to supergroup B) is indicated as recombinant between the corresponding *ori *sequences of *w*Mel_A (A-supergroup) and *w*Ma_B (B-supergroup) strains. A similar recombination event, but among supergroup B strains, is observed in *w*Calt_B, which appears to be recombinant between *w*Osca_B and *w*Vul_B.

Significant recombination events at the *ori *region of *Wolbachia *were detected within both supergroup A and B and between the two supergroups (MaxChi, P < 0.001). Most of the recombination breakpoints fell at one of the two edges of the intergenic region (see graph in Figure 3A). The majority of binding sites (excluding DnaA_2 and CtrA_2) occur within a region that experienced a similar low number of recombination events, suggesting that usually these sites, when recombining, are exchanged as a unique sequence tract. A likely recombination breakpoint occurred in the nucleotide region between IHF_1 and DnaA_2 (see arrow, Fig. 3A) indicating a potential shuffling among binding sites at this region.

Most of the recombination breakpoints per sequence were single and thus involved recombination of sequences encompassing one of the two halves of the alignment. Clear examples of recombinant tracts can be visualized by simple examination of the shared nucleotide polymorphisms among triplets of sequences (see example in Figure 3B). For instance, the *ori *region of *w*Aenc_B, a strain from the host *A. encedon *belonging to supergroup B, shows a clear recombinant pattern between A- and B-type sequences. Specifically, the first 210 bp of the sequence from the *w*Aenc_B strain shares most of the polymorphisms (70) with the B strain *w*Ma_B, from *D. simulans*, while the remaining sequence portion shares most of polymorphisms (42) with the A strain *w*Mel-A, from *D. melanogaster*. As a result, *w*Aenc_B places as a deep branch of supergroup B, separated by a great distance from other B-strains (bootstrap value, P = 82; data not shown); this apparent phylogenetic divergence is actually due to recombination within the sequence. A similar case of recombination is found for the *w*Calt_B strain and involves B-type sequences. The first 500 bp of the *w*Calt_B sequence shares most of the polymorphisms with the *w*Vul sequence, while the following 173 bp share most polymorphisms with the *w*OscaB sequence. Other instances of recombination involve the *Wolbachia *A-strains from *A. albopictus, A. sparsa, C. pennsylvanicus *and S. *invicta*, denoted A2 in figure 2. These four *ori *sequences are recombinant between A and B-types sequences (MaxChi, P < 0.001).

## Discussion

In the present study, we provide *in silico *evidence for the location of the origin of replication in *Wolbachia *and its close relatives: *Ehrlichia*, *Anaplasma*, *Rickettsia *and *Neorickettsia*. The analysis included fifty-three *Wolbachia *strains and ten additional strains from the other closely related bacterial species. All the origins predicted here are 383–620 bp long. The *Ehrlichia*, *Anaplasma*, *Neorickettsia*, and *Rickettsia *predicted origins herein do align appropriately with the shift of GC-skew found in these genomes [36]. However, the actual shift occurs over a fairly large region (~20 kB) and thus is not sufficient for identifying the precise *ori*. The *Wolbachia *genomes have no clear shift in GC-skew which could indicate a putative origin of replication [14,15,17]. The fact that *Wolbachia *genomes do not present a strong shift of GC-skew may be due to extensive intragenomic recombination events that in addition may have also eliminated the synteny between the genomes of the genes flanking the *ori*, another feature of the *Wolbachia *lineage. Recombination has been recently shown to be widespread across *Wolbachia *genomes [62]. Based on our analyses, it has clearly played a role in shaping the *ori *region of *Wolbachia *and potentially shuffled the binding sites, thus giving rise to chimeric sequences. Whether and how these DNA rearrangements have affected the replication performance in the recombinant strains remains a subject of future investigation.

### Prediction of origins in genomes

As a non-coding region, the *ori *region is often overlooked and not annotated in genomes. However, this is a significant issue since it has an essential role in DNA replication. Therefore, we have established several criteria that should be used to identify the origin from genome sequencing data including: (a) boundary genes that are homologous to those of closely related bacteria, (b) an intergenic region 200–1000 bp in size, (c) presence of appropriate binding sites (primarily for DnaA and IHF), (d) an increased distribution of the appropriate binding sites when compared to other intergenic regions, (e) increased AT content relative to the genome, (f) increased homology to closely related sequences relative to the genome, and (g) a shift in GC-skew. Although all seven elements may not be present in all genomes, a significant combination of these elements should allow for a better prediction of this important region.

### The origin of replication and the CtrA, DnaA and IHF binding sites

The presumed *ori *regions of the bacterial species and strains of the present study are characterized by the presence of DnaA, CtrA and IHF boxes (the exception being *N. sennetsu*). The DnaA and IHF boxes are present in all bacterial *ori *regions characterized. However, the CtrA boxes seem to be restricted, as yet, to the *ori *regions of the α-Proteobacteria. In *C. crescentus*, CtrA is a global cell cycle regulator [26] and more specifically, the response regulator protein of a two-component signal transduction signal. Upon phosphorylation by a sensor histidine kinase, CtrA binds to its corresponding binding sites and represses chromosomal replication [63]. CtrA's binding sites are overlapping with a DnaA box and the IHF binding site. Thus, binding of CtrA prevents binding of DnaA and IHF [37]. CtrA is degraded before the onset of the S phase by the protease ClpXP allowing DnaA and IHF binding [64,65]. Despite *A. marginale *CtrA having 59% amino acid identity with its *w*Mel ortholog, *A. marginale *and *A. phagocytophilum ori *regions have only a single CtrA binding site, as opposed to four in *w*Mel. *E. ruminantium *has no CtrA binding site in its putative *ori *region while its CtrA protein is 80% identical to the *A. marginale *ortholog. Similarly, the putative *ori *regions of the pseudoscorpion *C. scorpioides Wolbachia *strain, *E. chaffeensis*, *E. canis*, *R. conorii *and the filarial nematode *B. malayi Wolbachia *strain (*w*Bm) have zero, one, three, two and two CtrA binding sites respectively. These observations suggest that CtrA may be dispensable in these bacteria. Interestingly, the bacteria with zero to three CtrA binding sites in their *ori *region are not associated with insects. In contrast, insect-associated *Wolbachia *and *Rickettsia *bacteria usually present high number of CtrA binding sites present in their *ori *region ranging from four to seven (the exception being *R. felis *which has three CtrA binding sites). Whether bacterial growth control is host-dependent and is regulated through the number of the CtrA binding sites in the origin of replication region awaits experimental confirmation.

It is worth noting that there are orthologs to *dnaA*, *ctrA *and *ihfAB *in the bacterial genomes studied except *N. sennetsu *and *A. marginale*. They are both missing IHF-β but have retained the IHF-α subunit [see Additional Table 3]. Another interesting observation is that the filarial nematode *B. malayi Wolbachia *strain (*w*Bm) and *A. phagocytophilum *are missing both *parA *and *parB*, which are involved in partitioning the chromosomes [14,36]. This suggests that *Wolbachia *and *Anaplasma *replication may be quite interesting; experimental work is needed in order to clarify the roles of replication-associated proteins in binding the *ori *and initiating DNA replication initiation process.

IHF binding sites were found in all bacteria using the consensus WATCAN_5_WTR [37]. In some *Wolbachia *strains, two to three such candidate sites were found within their putative *ori *regions. In these cases, only the common one was retained [37]. In contrast, *A. marginale *and *A. phagocytophilum *had different IHF binding sites in both in sequence and in position. Two conserved putative IHF binding sites were detected in the putative *ori *region of *E. canis *and the two *E. ruminantium *strains, with all of them being similar both in sequence and position while a single putative IHF binding site was present in *E. chaffeensis *which was the only common IHF binding site found in all four *Ehrlichia *strains. IHF positioning varied between the four *Rickettsia *species. The only exception appears to be the conservation in position as well as in sequence between *R. prowazekii *and *R. typhi*. The IHF-β subunit is missing from the genome of *A. marginale*. The only IHF identified, AM_006, is 43.2% identical (amino acid level) to the *w*Mel IHF-α subunit (WD_0057). *A. phagocytophilum*, all four *Ehrlichia*, and all four *Rickettsia *species have both IHF subunits. *N. sennetsu *also lacks IHF-β [see Additional Table 3]. Whether the absence of the IHF-β subunit is somehow correlated with the divergence/absence of IHF binding sites in *Anaplasma *and *Neorickettsia *is not known.

### The evolution of the origin of replication of *Wolbachia*, *Ehrlichia*, *Anaplasma*, *Rickettsia *and *Neorickettsia*

The difference in the boundary genes observed between the closely related *Anaplasma*, *Ehrlichia*, *Wolbachia *and the other α-Proteobacteria is likely due to chromosomal rearrangements that have taken place in the *ori *region. All of the origins examined have as boundaries either the COG1253 – *hemE *pair (*Wolbachia*, *Ehrlichia *and *Anaplasma*), or the *hemE *– COG1806 pair (*Caulobacter *and *Rickettsia*), or the *hemE *– uncharacterized phage protein pair (*N. sennetsu*). Overlaying these observations on the phylogeny of the α-Proteobacteria [36], the most likely ancestral configuration is that found in *Caulobacter *and *Rickettsia*. Sometime after the branching of the *Anaplasmataceae*, a rearrangement took place that repositioned the COG1253 gene to the position of COG1806 orthologs in *Wolbachia*, *Ehrlichia*, and *Anaplasma *with a second rearrangement occurring in *N. sennetsu*. The exact nature of these rearrangements cannot be determined since the synteny of the chromosomes has been lost. As discussed earlier, the distribution of *ori*-specific binding sites (DnaA, CtrA and IHF) is consistent with the currently accepted phylogeny of *Wolbachia*.

### Growth control, infection levels and *Wolbachia*-induced phenotypes

It has been widely accepted that bacterial infection levels are positively correlated with the virulent and/or pathogenic properties of bacterial pathogens [66]. Similarly, several studies have shown that *Wolbachia*'s ability to induce reproductive and/or virulent phenotypes is positively correlated with their intra-host infection levels [11-13,54,67]. Furthermore, intracellular bacteria that are transmitted through the eggs of their hosts must be "prudent replicators" in order to ensure their transmission to future generations by preventing overgrowth that could lead to host death prior to reproduction. This raises the question: is the DNA replication and growth of an obligate intracellular bacterium under bacterial control, host control, phage control or some combination? Overreplication by the "popcorn" strain of *Wolbachia *in *D. melanogaster *suggests that the bacterial strain has a strong influence on replication rates within the host [54], although effects of the host also occur [12] and effects of phage have not been tested [10].

The results of the present study clearly indicate that no major differences could be detected between the *ori *regions of *Wolbachia *strains differing in ability to induce CI, virulence, parthenogenesis or feminization. All *Wolbachia *strains presented the same organization in their putative origin of replication: COG1253 and *hemE *as boundary genes, three DnaA boxes, two to seven CtrA boxes and a single IHF binding site. A notable exception is the pseudoscorpion *Wolbachia *strain which presents zero CtrA binding sites. The possibility that DNA binding specificity of CtrA has changed in these bacteria is an intriguing hypothesis. CtrA contains two domains, a receiver domain at the N-terminus and a transcriptional regulatory (DNA binding) effector domain at the C-terminus. These domains are nearly identical between *w*Mel and *w*Bm with only one positive amino acid substitution in each domain. All other changes are located at the far C-terminus where no domains are found. Since a single amino acid change can greatly affect DNA binding, it is conceivable that these two positive substitutions can alter CtrA's binding specificity. A second hypothesis is that CtrA's DNA-binding specificity did not change in *w*Bm, and only the number of CtrA binding sites reduced. This CtrA binding site reduction may be associated with the mutualistic nature of the *Wolbachia *– *B. malayi *association and may have resulted in a novel control of bacterial replication. These hypotheses need to be tested experimentally.

### Origin of replication and genetic transformation system

Evidence presented here for the prediction of *ori *location, is based on the boundary genes and on the *in silico *finding of characteristic DnaA, CtrA, and IHF binding sites that have been experimentally confirmed only in *C. cresentus *and partially in *R. prowazekii *[37]. Lack of a robust genetic transformation system for *Wolbachia*, *Ehrlichia*, *Neorickettsia *and most *Anaplasma *and *Rickettsia *precludes experimental verification in the bacterial species of the present study. However, significant progress has been made toward the development of a robust genetic transformation for *A. phagocytophilum *and *R. prowazekii *using homologous recombination-based and transposon-based approaches [68-70]. The fact that these bacteria (both *Wolbachia *and relatives) can be maintained in different cell lines [3,71,72], the availability of complete genomic information [14,15,34-36,73-76] and, the presence in some of them, such as *Wolbachia*, of endogenous phages and insertion sequences [77,78] will certainly facilitate current efforts for the genetic transformation of these intracellular bacteria.

## Conclusion

We provide *in silico *evidence for the location of the origin of replication in *Wolbachia *and its close relatives: *Ehrlichia*, *Anaplasma*, *Rickettsia *and *Neorickettsia*. Putative origins of replication, which are usually 200–1000 bp long, have features in common that were used to establish a set of guidelines for properly predicting the origin in genomes. Several of the bacteria had variable sequences/numbers of key binding sites suggesting altered modes of replication. This is supported by the lack of specific replication associated enzymes.

Intracellular bacteria that are transmitted through the eggs of their hosts are thought to be "prudent replicators" in order to ensure their transmission to future generations by not causing lethality of their host prior to reproduction. This raises the question: is the DNA replication and growth of an obligate intracellular bacterium under bacterial control, host control, phage control or some combination? The results of the present study clearly indicate that no major differences could be detected between the *ori *regions of *Wolbachia *strains inducing or non-inducing CI, being virulent or non-virulent, inducing parthenogenesis or feminization. In addition, recombination across the *ori *region was demonstrated for the main arthropod supergroups, A and B. Lastly and surprisingly, the origin boundary genes have changed twice in the evolution of this order of bacteria.

## Methods

### DNA extraction

Various insect hosts and *Wolbachia *strains were used for DNA extraction in the present study [see Additional Table 1]. Fly stocks were reared on standard corn flour – sugar – yeast medium at 25°C. Bacterial DNA was extracted using the DNeasy Tissue Kit (Qiagen) according to the manufacturer's instructions.

### PCR amplification and sequencing of the *Wolbachia ori *region

A PCR strategy [see Results, Additional Figure 1 and Additional Table 2] was developed to amplify the *ori *region from the following *Wolbachia *strains belonging to supergroup A and B: *w*Mel, *w*MelPop, *w*Au, *w*Ri, *w*Ha, *w*Yak, *w*Tei, *w*San, *w*No, *w*Ma and *w*Mau. Various PCR primers were used for the amplification reactions [see Additional Table 1]. PCR reaction mixtures contained 1× Taq buffer (750 mM Tris-Cl pH 8.8, 200 mM (NH_4_)_2_SO_4_, 0.1% Tween 20), 2 mM MgCl_2_, 125 μM dNTPs, 12.5 pmol of each primer, 1 unit Taq polymerase (Promega) and 25 ng/μl template DNA and were cycled with an initial denaturing step at 94°C for 10 minutes, 35 cycles of 94°C for 30 seconds, 54°C for 30 seconds and 72°C for 3 minutes followed by a final extension at 72°C for 10 minutes. PCR reactions were purified with Qiagen nucleotide removal kit or Qiagen gel extraction kit depending on the existence or not, of byproducts. Sequencing was performed by Macrogen (Korea). Sequence trace files from sequencing reactions were processed using the DNAStar 5.0 suite of programs.

A smaller region of *ori *was amplified and sequenced from a greater variety of *Wolbachia *strains according to the methods mentioned in [79]. Reactions to generate these smaller amplicons were attempted for 52 *Wolbachia *strains from almost all described supergroups using standard PCR conditions with HotStarTaq (Qiagen), according to the manufacturer's suggestions, with 0.5 μM of each WD_1027_R and WD_1028_R. Reactions were initiated with a 15 minute incubation at 95°C followed by 50 cycles of 95°C for 30 seconds, 55°C for 30 seconds, 72°C for 1 min. The primers WD_1027_R and WD_1028_R had 5'-tags of M13 forward and reverse primer sequences, respectively, to serve as anchors for the degenerate primers in later stages of amplification and were later used for sequencing. Amplification reactions (8 μL) were treated with 0.5 U shrimp alkaline phosphatase and 1.0 U exonuclease I (Amersham) in the supplied buffer. Sequencing reactions were performed at the J. Craig Venter Joint Technology Center (Rockville, MD) with M13 forward and reverse primers. Assembly was done with the TIGR assembler and manually curated with Cloe (ClosureEditor, a TIGR program for editing assemblies). The alignment was initially generated using CLUSTALW and curated in Bioedit v. 7.0.4.1.

The nucleotide sequences reported in this study have been deposited in GenBank under accession numbers DQ498834 – DQ498882.

### Sequence acquisition and analysis from publicly available genomes

Sequence information was used from publicly available genome data (GenBank) of *Wolbachia *and other closely related bacterial species and strains (Table 1).

### *In silico *prediction of the *ori *region

For *ori*-boundary identification, genes homologous to COG1253 (see results for the new proposed gene name of the CBS domain protein) and *hemE *were identified using BLAST. Moreover, the CBS domain protein gene was searched for conserved domains using the HMMer program [41] and the Pfam_fs hidden markov model (HMM) database.

Binding sites were identified using local perl scripts based on the previously determined consensus sequences [37]. For the CtrA binding site (TTAA-N_7_-TTAA), a single mismatch was allowed only in the A's and only if these mismatches followed the looser consensus TTWW-N_7_-TTWW described previously [37]. For the DnaA binding site, the consensus TTATNCACA was used [80]. For the IHF-binding site, the consensus WATCAN_5_WTR was used [81,82].

In order to examine the distribution of the CtrA-, IHF-, and DnaA-binding sites in the intergenic regions of each genome were identified using fuzznuc from the EMBOSS package [83] and locally developed scripts.

### Phylogenetic analysis

The alignment for the figure 2 tree was generated using ClustalX 1.83 for Windows [84] with the default parameters and trimmed using BioEdit for Windows [85]. Maximum likelihood (ML) methods were used to infer phylogenetic relationships. Prior to ML analysis, a DNA substitution model was selected using Modeltest v3.06 and the Akaike information criterion (AIC). The selected model of evolution was based on a 382 bp *ori *region with indels excluded (TVM+G). ML heuristic searches were performed using 100 random taxon addition replicates with tree bisection and reconnection (TBR) branch swapping. ML bootstrap support was determined using 100 bootstrap replicates, each using 10 random taxon addition replicates with TBR branch swapping. Searches were performed in parallel on a Beowulf cluster using a clusterpaup program and PAUP version 4.0b10 [86].

Conservation of binding domains in the *Wolbachia ori *region was investigated within three clades of closely related *Wolbachia *(B1, B2 and A1). It should be noted here that the A2 clade (see Figure 2) was not used because it is recombinant between the A and B supergroups in the *ori *region (see text for details). Binding site positions were identified based on a reference strain within each clade; this was straightforward due to relatively low variation within each clade. Positions were then classified as binding or non-binding. Positions within binding domains with free nucleotide designations (N) were classified as non-binding sites. In cases where binding domains were overlapping, if both positions were N sites, then the position was classified as non-binding, otherwise it was classified as a binding position. All positions within the IGR were then scored as polymorphic (containing at least one polymorphism) or not. Fisher Exat Tests were used to compare the proportions of polymorphic and non-polymorphic positions in binding sites versus non-binding sites.

### Recombination analyses

The *ori *region of *Wolbachia *was searched for recombination signature by using the Maximum Chi Square (MaxChi2) program, implemented in the RDP2 program [87]. MaxChi is a local method that uses a sliding window approach to search for putative recombination breakpoints in a set of aligned DNA sequences. Significant discrepancies between the two partitions of the window are calculated based on the difference in the number of variable sites (VI) on either sides of the central partition. A Chi-square statistics is applied. The step size was set to 20 nucleotides and the window size set to 20 VI, gaps were included and a Bonferroni correction was applied. The highest acceptable P-value cut-off was set to 0.001 and 1000 permutation were generated. We analyzed only strains belonging to supergroup A and B and having sequences of equal length encompassing a portion of both flanking genes. The final alignment was 673 base pairs long and included 38 strains (19 A- and 19 B-strains). The alignment was partitioned in dataset A and B (from the two supergroups) and recombination analyses were run on both single and combined datasets.

## Abbreviations

*ori*: origin of DNA replication

CBS: Cystathione-beta-synthase domain

IHF: Integration Host Factor

IGR: Intergenic Region

## Authors' contributions

PI designed primers, amplified and analyzed sequences, carried bioinformatics analysis and drafted the manuscript.

JCDH amplified and analyzed sequences across diverse *Wolbachia *strains, carried genomics and bioinformatics analysis, and drafted portions of the manuscript

PS designed primers, amplified and analyzed sequences across diverse *Wolbachia *strains.

SS amplified and analyzed sequences across diverse *Wolbachia *strains.

GT amplified and analyzed sequences across diverse *Wolbachia *strains.

SRB designed primers, carried out the phylogenetic analysis and drafted the corresponding portion of the manuscript.

LB carried out the recombination analysis and drafted the corresponding portion of the manuscript.

JHW participated in the design of the study and coordination of research.

KB conceived of the study, participated in its design and coordination and drafted the manuscript.

All authors read and approved the final manuscript.

## Supplementary Material

Additional file 1 **Additional Figure 1 **– PCR strategy followed for amplifying and sequencing the *ori *region along with the two flanking genes of different *Wolbachia *strains. FRAF1 is either A-group (FRAF1MEL) or B-group (FRAF1PIP) specific and binds in slightly different positions. Click here for file

Additional file 2 **Additional Figure 2 **– The Maximum Likelihood derived trees demonstrate a clear concordance of the *ori *region and 16S *rRNA *phylogenies for the *Rickettsia *genus (Figure 2C), *Anaplasma/Ehrlichia *genera (Figure 2B), and *Wolbachia *supergroups with one minor exception (Figure 2A). There is a weakly supported trifurcation in the 16S *rRNA *phylogeny (spanning supergroups A, B, and E, 64% bootstrap support) that is inconsistent with the *ori *phylogeny (98% bootstrap support). Supergroups A, B, D, E, and F are represented by sequences from *D. melanogaster*, *C. pipiens*, *B. malayi*, *F. candida*, and *C. lectularius*, respectively. The likelihood-based Shimodaira-Hasegawa (SH) test for alternative tree topologies [90] however revealed no significant difference between any of the Figure 2 topologies, specifying the *ori *region as a sufficient genetic marker for the broad evolutionary relationships in this intracellular clade. Selected DNA substitution models of evolution were selected using Modeltest v3.06 and the Akaike information criterion (AIC). They were based on a 655 bp *ori *region (TVM+I) and 1377 bp 16S *rRNA *sequence (HKY) for *Wolbachia*, a 395 bp *ori *region (GTR+I) and 1454 bp 16S *rRNA *sequence (GTR+I) for the *Anaplasma/Ehrlichia *genera, and a 382 bp *ori *region (HKY+I) and 1498 bp 16S *rRNA *sequence (TrN) for *Rickettsia*. ML heuristic searches were performed using 500 random taxon addition replicates with tree bisection and reconnection (TBR) branch swapping. ML bootstrap support was determined using 500 bootstrap replicates, each using 10 random taxon addition replicates with TBR branch swapping. Searches were performed in parallel on a Beowulf cluster using a clusterpaup program and PAUP version 4.0b10 [86]. Click here for file

Additional file 3 **Additional Table 1 **– *Wolbachia *strains and closely related bacterial species used in this work. Click here for file

Additional file 4 **Additional Table 2 **– Primers used in this study. Click here for file

Additional file 5 **Additional Table 3 **– Replication enzymes in complete genomes. Click here for file

Additional file 6 **Additional Text File 1 **– Consensus sequence of the *ori *region based on which the PCR strategy presented in Additional Figure 1 and Additional Table 3 was developed. Click here for file
