# Supplementary figures and images for: New criteria for selecting the origin of DNA replication in Wolbachia and closely related bacteria
Source: BMC Genomics. 2007 Jun 20;8:182. doi: 10.1186/1471-2164-8-182 (PMC1914354; doi:10.1186/1471-2164-8-182)

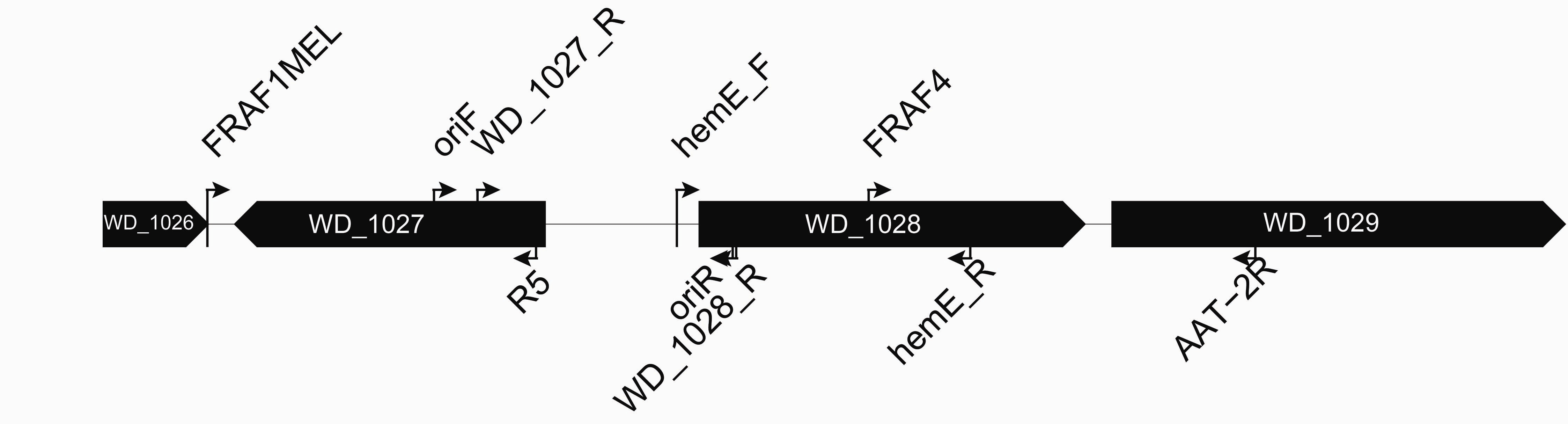

Supplement: Additional file 1 — Additional Figure 1 – PCR strategy followed for amplifying and sequencing the ori region along with the two flanking genes of different Wolbachia strains. FRAF1 is either A-group (FRAF1MEL) or B-group (FRAF1PIP) specific and binds in slightly different positions. [file 1471-2164-8-182-S1.jpeg]

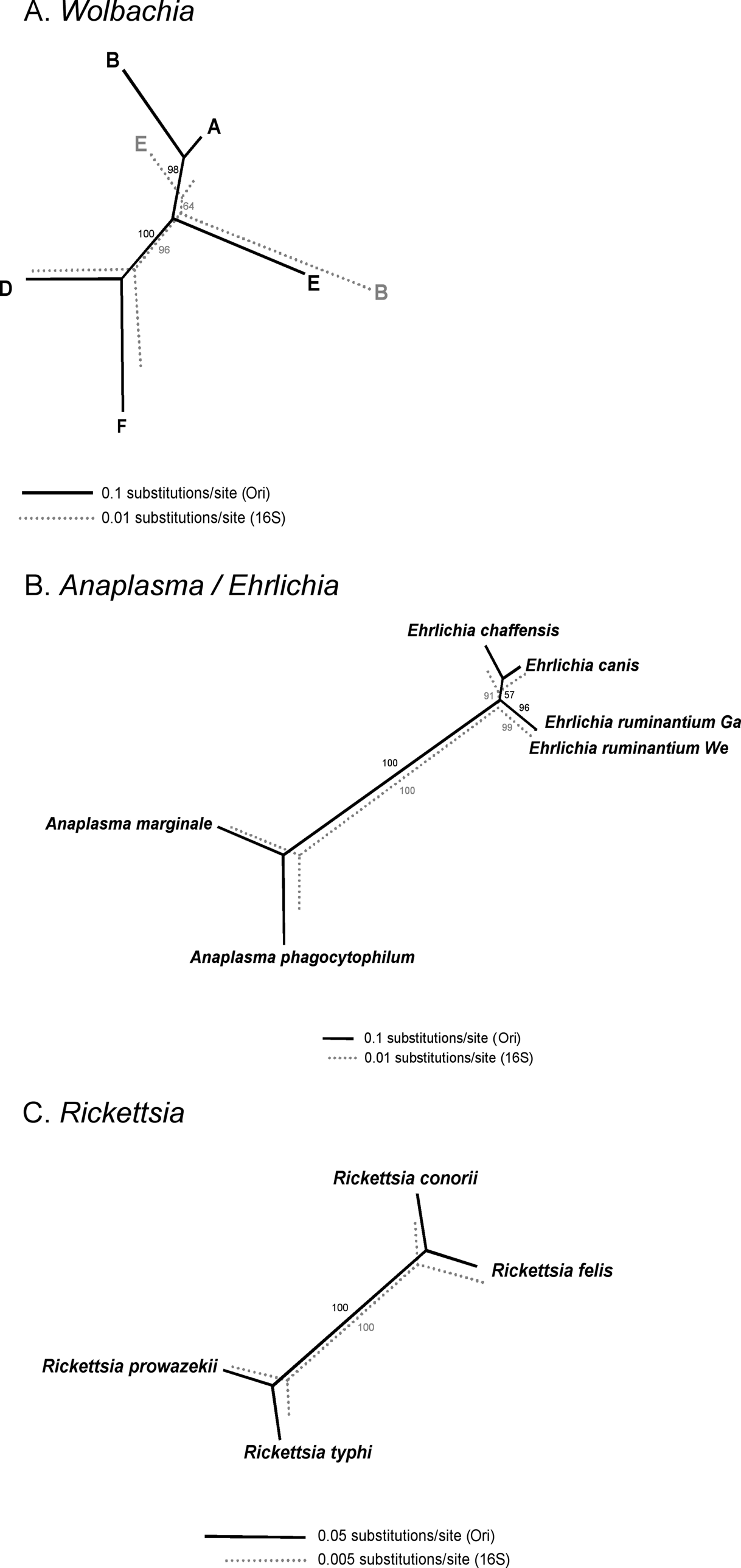

Supplement: Additional file 2 — Additional Figure 2 – The Maximum Likelihood derived trees demonstrate a clear concordance of the ori region and 16S rRNA phylogenies for the Rickettsia genus (Figure 2C), Anaplasma/Ehrlichia genera (Figure 2B), and Wolbachia supergroups with one minor exception (Figure 2A). There is a weakly supported trifurcation in the 16S rRNA phylogeny (spanning supergroups A, B, and E, 64% bootstrap support) that is inconsistent with the ori phylogeny (98% bootstrap support). Supergroups A, B, D, E, and F are represented by sequences from D. melanogaster, C. pipiens, B. malayi, F. candida, and C. lectularius, respectively. The likelihood-based Shimodaira-Hasegawa (SH) test for alternative tree topologies [90] however revealed no significant difference between any of the Figure 2 topologies, specifying the ori region as a sufficient genetic marker for the broad evolutionary relationships in this intracellular clade. Selected DNA substitution models of evolution were selected using Modeltest v3.06 and the Akaike information criterion (AIC). They were based on a 655 bp ori region (TVM+I) and 1377 bp 16S rRNA sequence (HKY) for Wolbachia, a 395 bp ori region (GTR+I) and 1454 bp 16S rRNA sequence (GTR+I) for the Anaplasma/Ehrlichia genera, and a 382 bp ori region (HKY+I) and 1498 bp 16S rRNA sequence (TrN) for Rickettsia. ML heuristic searches were performed using 500 random taxon addition replicates with tree bisection and reconnection (TBR) branch swapping. ML bootstrap support was determined using 500 bootstrap replicates, each using 10 random taxon addition replicates with TBR branch swapping. Searches were performed in parallel on a Beowulf cluster using a clusterpaup program and PAUP version 4.0b10 [86]. [file 1471-2164-8-182-S2.jpeg]
