# Supplementary material for: New criteria for selecting the origin of DNA replication in Wolbachia and closely related bacteria
Source: BMC Genomics. 2007 Jun 20;8:182. doi: 10.1186/1471-2164-8-182 (PMC1914354; doi:10.1186/1471-2164-8-182)
Supplement: Additional file 3 — Additional Table 1 – Wolbachia strains and closely related bacterial species used in this work. [file 1471-2164-8-182-S3.doc]

**Supplementary Table 1. *Wolbachia*** strains and closely related bacterial species used in this work

| Bacterium (strain^a^) | *Wolbachia* host | *Wolbachia* supergroup | Number of binding sites | | |
| --- | --- | --- | --- | --- | --- |
|  |  |  | DnaA | CtrA | IHF |
| *w*AspaA | *Acromis sparsa* | A | 3 | 5 | 1 |
| *w*AlbA | *Aedes albopictus* | A | 3 | 6 | 1 |
| *w*CpenA | *Camponotus pennsylvanicus* | A | 3 | 5 | 1 |
| *w*Ana | *Drosophila ananassae* | A | 3 | 4 | 1 |
| *w*DbifA | *Drosophila bifasciata* | A | 3 | 4 | 1 |
| *w*Din | *Drosophila innubila* | A | 3 | 4 | 1 |
| *w*Mel | *Drosophila melanogaster* | A | 3 | 4 | 1 |
| *w*MelPop | *Drosophila melanogaster* | A | 3 | 4 | 1 |
| *w*DneoA | *Drosophila neotestacea* | A | 3 | 4 | 1 |
| *w*DoriA | *Drosophila orientacea* | A | 3 | 4 | 1 |
| *w*DrecA | *Drosophila recens* | A | 3 | 4 | 1 |
| *w*San | *Drosophila santomea* | A | 3 | 4 | 1 |
| *w*Au | *Drosophila simulans* | A | 3 | 4 | 1 |
| *w*Ri | *Drosophila simulans* | A | 3 | 4 | 1 |
| *w*Ha | *Drosophila simulans* | A | 3 | 4 | 1 |
| *w*Sim | *Drosophila simulans* | A | 3 | 4 | 1 |
| *w*Tei | *Drosophila teissieri* | A | 3 | 4 | 1 |
| *w*Wil | *Drosophila willistoni* | A | 3 | 4 | 1 |
| *w*Yak | *Drosophila yakuba* | A | 3 | 4 | 1 |
| *w*KueA | *Ephestia kuehniella* | A | 3 | 4 | 1 |
| *w*Mors | *Glossina morsitans* | A | 3 | 4 | 1 |
| *w*Uni | *Muscidifurax uniraptor* | A | 3 | 4 | 1 |
| *w*Ngir | *Nasonia giraulti* | A | 3 | 4 | 1 |
| *w*Nlon | *Nasonia longicornis* | A | 3 | 4 | 1 |
| *w*NvitA | *Nasonia vitripennis* | A | 3 | 4 | 1 |
| *w*PhofA | *Pegoscapus hoffmeyeri* | A | 3 | 3 | 1 |
| *w*SinvictaA | *Solenopsis invicta* | A | 3 | 5 | 1 |
| *w*AencB | *Acraea encedon* | B | 3 | 4 | 1 |
| *w*AepoB | *Acraea eponina* | B | 3 | 6 | 1 |
| *w*AsocconB | *Allonemobius socius* | B | 3 | 5 | 1 |
| *w*AsocB | *Allonemobius socius* | B | 3 | 5 | 1 |
| *w*Vul | *Armadillidium vulgare* | B | 3 | 7 | 1 |
| *w*Calt | *Chelymorpha alternans* | B | 3 | 5 | 1 |
| *w*Pip | *Culex pipiens* | B | 3 | 6 | 1 |
| *w*Pip | *Culex pipiens quinquefasciatus* | B | 3 | 6 | 1 |
| *w*Mau | *Drosophila mauritiana* | B | 3 | 5 | 1 |
| *w*Ma | *Drosophila simulans* | B | 3 | 5 | 1 |
| *w*No | *Drosophila simulans* | B | 3 | 5 | 1 |
| *w*For | *Encarsia formosa* | B | 3 | 6 | 1 |
| *w*KueB | *Ephestia kuehniella* | B | 3 | 6 | 1 |
| *w*GfirB | *Gryllus firmus* | B | 3 | 5 | 1 |
| *w*NvitB | *Nasonia vitripennis* | B | 3 | 5 | 1 |
| *w*OscaB | *Ostrinia scapulalis* | B | 3 | 5 | 1 |
| *w*ProtB | *Protocalliphora sialia* 00172 | B | 3 | 6 | 1 |
| *w*Con | *Tribolium confusum* | B | 3 | 6 | 1 |
| *w*Dei | *Trichogramma deion* | B | 3 | 5 | 1 |
| *w*KayB | *Trichogramma kaykai* | B | 3 | 5 | 1 |
| *w*TaiB | *Teleogryllus taiwanemma* | B | 3 | 5 | 1 |
| *w*UrtB | *Tetranychus urticae* | B | 3 | 6 | 1 |
| *w*Bm | *Brugia malayi* | D | 3 | 2 | 1 |
| *w*FcanE | *Folsomia candida* | E | 3 | 5 | 1 |
| *w*ClecF | *Cimex lectularius* | F | 3 | 2 | 1 |
| *w*Csco | *Cordylochernes scorpioides* | No grouping | 3 | 0 | 1 |
| *Anaplasma marginale* St. Maries | N/A | N/A | 2 | 1^b^ | 1 |
| *Anaplasma phagocytophilum* HZ | N/A | N/A | 4 | 1 | 1^c^ |
| *Ehrlichia ruminantium* Welgevonden^d^ | N/A | N/A | 2 | 0^e^ | 2 |
| *Ehrlichia canis* Jake | N/A | N/A | 4 | 3 | 2 |
| *Ehrlichia chaffensis* Arkansas | N/A | N/A | 4 | 1 | 1 |
| *Rickettsia prowazekii* Madrid E | N/A | N/A | 4 | 6 | 1 |
| *Rickettsia conorii* Malish 7 | N/A | N/A | 3 | 2 | 1^c^ |
| *Rickettsia typhi* Wilmington | N/A | N/A | 4 | 5 | 1 |
| *Rickettsia felis* URRWXCal2 | N/A | N/A | 3 | 3 | 1 |
| *Neorickettsia sennetsu* Miyayama | N/A | N/A | 0 | 0 | 0 |
|  | N/A | N/A | 0 | 0 | 2 |
| *Caulobacter crescentus* CB15^f^ | N/A | N/A | 5 | 5 | 1 |

N/A: not applicable.

CtrA binding site consensus: TTAA-N_7_-TTAA with one mismatch in the A’s

DnaA box consensus: TTAΤNCACA

IHF binding site consensus: WATCAN_5_WTR

^a^ The first 53 strains are found in *Wolbachia*.

^b^ The single CtrA binding site of *A. marginale* is located 150 nucleotides downstream COG1253’s start codon

^c^ IHF binding site falls inside *hemE* (for *A. phagocytophilum*) or inside COG1806 (for *R. conorii*).

^d^ *E. ruminantium* Welgevonden is identical to *E. ruminantium* Gardel and is not shown separately in this table.

^e^ two CtrA boxes can be found if two mismatches are permitted.

^f^ Protein binding sites of *C. crescentus* *ori* region were taken from the manuscript which characterized it experimentally (Brassinga et al. 2002). These binding sites are not necessarily located between *hemE* and COG1806 genes.
