# Supplementary material for: New criteria for selecting the origin of DNA replication in Wolbachia and closely related bacteria
Source: BMC Genomics. 2007 Jun 20;8:182. doi: 10.1186/1471-2164-8-182 (PMC1914354; doi:10.1186/1471-2164-8-182)
Supplement: Additional file 4 — Additional Table 2 – Primers used in this study. [file 1471-2164-8-182-S4.doc]

**Supplementary Table 2**. Primers used in this study

| Primer name | Sequence |
| --- | --- |
| FRAF1MEL | tttaactataacttgagttgcata |
| FRAF1PIP | ttaagaaattgtttaaattgcata |
| oriF | AGCCTATTACGTTRTCAAAGT |
| hemE_F | TAACAAKTTATTGATAGAAC |
| FRAF4 | TGGACRGTAGCYTCMTACATCATA |
| R5 | GAACTTTAGTAGAAAAAATAGCATA |
| oriR | AAGAGACCTRCCAGCCTGA |
| hemE_R | TTGGAAAACCTATTATTGGA |
| AAT-2R | TCAAYRTAYGARACCCAATAAG |
| WD1027_R | CYKTACGTGGAGTCATTATRTC |
| WD1028_R | GCGRTAYTCAGGAAGAGAYCTRCC |
