# Supplementary material for: New criteria for selecting the origin of DNA replication in Wolbachia and closely related bacteria
Source: BMC Genomics. 2007 Jun 20;8:182. doi: 10.1186/1471-2164-8-182 (PMC1914354; doi:10.1186/1471-2164-8-182)
Supplement: Additional file 5 — Additional Table 3 – Replication enzymes in complete genomes. [file 1471-2164-8-182-S5.doc]

**Supplementary Table 3 – Presence of DnaA, CtrA and IHF genes in complete bacterial genomes**

| Organism | DnaA | CtrA | IHF subunits^1^ |
| --- | --- | --- | --- |
| *Wolbachia pipientis w*Mel | WD_0001 | WD_0732 | WD_0057; WD_0065; WD_1089 |
| *Wolbachia pipientis w*Bm | Wbm_0276 | Wbm_0596 | Wbm_0661; Wbm_0572; Wbm_0058 |
| *Ehrlichia chaffeensis* Arkansas | ECH_0809 | ECH_1012 | ECH_0804; ECH_0162; ECH_0400 |
| *Ehrlichia ruminantium* Welgevonden | Erum_2870 | Erum_7860 | Erum_1080; Erum_2930; Erum_6140 |
| *Ehrlichia ruminantium* Gardel | ERGA_CDS_02880 | ERGA_CDS_08210 | ERGA_CDS_02930; ERGA_CDS_01010; ERGA_CDS_06360 |
| *Ehrlichia canis* Jake | Ecaj_0270 | Ecaj_0817 | Ecaj_0107; Ecaj_0274; Ecaj_0619 |
| *Anaplasma marginale* St. Maries | AM_430 | AM_1016 | AM_006 |
| *Anaplasma phagocytophilum* HZ | APH_0474 | APH_1099 | APH_1281; APH_0784; APH_0271 |
| *Neorickettsia sennetsu* Miyayama | NSE_0259 | NSE_0234 | NSE_0640 |
| *Rickettsia typhi* Wilmington | RT_0589 | RT_0061 | RT_0703; RT_0162; RT_0511 |
| *Rickettsia prowazekii* Madrid E | RP_601 | RP_071 | RP_708; RP_171; RP_524 |
| *Rickettsia conorii* Malish 7 | RC_0916 | RC_0101 | RC_0212; RC_1088; RC_0757 |
| *Rickettsia felis* URRWXCal2 | RF_0364, RF_0400 | RF_0056 | RF_0201; RF_1108; RC_0767 |
| *Caulobacter crescentus* CB15 | CC_0008 | CC_3035 | CC_1370; CC_3586; CC_2331; CC_1959 |

^1^ Based on the *Rickettsiales* Comparative database (protein clusters 696289 and 697661) and TIGRFAMs (TIGR00987 and TIGR00988).
